# A discussion on the robust vector autoregressive models: novel evidence from safe haven assets

**DOI:** 10.1007/s10479-022-04919-6

**Published:** 2022-08-18

**Authors:** Le Chang, Yanlin Shi

**Affiliations:** 1 Research School of Finance, Actuarial Studies and Statistics, Australian National University, Canberra, ACT 2601 Australia; 2 Department of Actuarial Studies and Business Analytics, Macquarie University, Sydney, NSW 2109 Australia

**Keywords:** Vector autoregressive model, Robust estimator, Safe haven assets, Realized volatility

## Abstract

The vector autoregressive (VAR) model has been popularly employed in operational practice to study multivariate time series. Despite its usefulness in providing associated metrics such as the impulse response function (IRF) and forecast error variance decomposition (FEVD), the traditional VAR model estimated via the usual ordinary least squares is vulnerable to outliers. To handle potential outliers in multivariate time series, this paper investigates two robust estimation methods of the VAR model, the reweighted multivariate least trimmed squares and the multivariate MM-estimation. The robust information criteria are also proposed to select the appropriate number of temporal lags. Via extensive simulation studies, we show that the robust VAR models lead to much more accurate estimates than the original VAR in the presence of outliers. Our empirical results include logged daily realized volatilities of six common safe haven assets: futures of gold, silver, Brent oil and West Texas Intermediate (WTI) oil and currencies of Swiss Francs and Japanese Yen. Our sample covers July 2017–June 2020, which includes the history-writing price drop of WTI on April 20, 2020. Our baseline results suggest that the traditional VAR model may significantly overestimate some parameters, as well as IRF and FEVD metrics. In contrast, robust VAR models provide more reliable results, the validity of which is verified via various approaches. Empirical implications based on robust estimates are further illustrated.

## Introduction

Ever since the 2008 global financial crisis, financial markets have become much more volatile, which holds true across various commodities, equities and currencies (see, for example, (Yaya et al., 2016; Ho et al., 2017; Hasannasab et al., 2019; Ma et al., 2019; Shi, 2020), among others). Many macro economic, social and political ‘black-swan’ events have caused historical-recording market turbulences, especially during recent periods. For instance, the Brexit and the US presidential election that took place in 2016 have introduced substantial co-movements in a wide range of global financial markets (Shaikh, 2017; Ameur & Louhichi, 2021). The recent pandemic, known as the COVID-19, has caused four circuit breakers within 10 days in March 2020 for the S &P 500 index (Ji et al., 2020; Spelta et al., 2021).

To mitigate such risks, especially for the more volatile equity markets, existing studies have pointed out the growing importance on allocating safe haven assets in one’s financial portfolio (see, for example, Baur and McDermott 2010; Flavin et al. 2014; Gurgun and Unalmis 2014; Baur and McDermott 2016, among others). In addition to the commonly used precious mental (e.g. gold and silver), recent literature indicates that crude oil and major currencies, such as Swiss Francs (CHF) and Japanese Yen (JPY), are also effective safe haven assets to hedge unforeseeable risks (Ranaldo & Söderlind, 2010; Ciner et al., 2013; Fatum & Yamamoto, 2016; Elie et al., 2019). However, even for those assets, black-swan events can still emerge, and the most recent one occurs in early-2020.

**Fig. 1:**
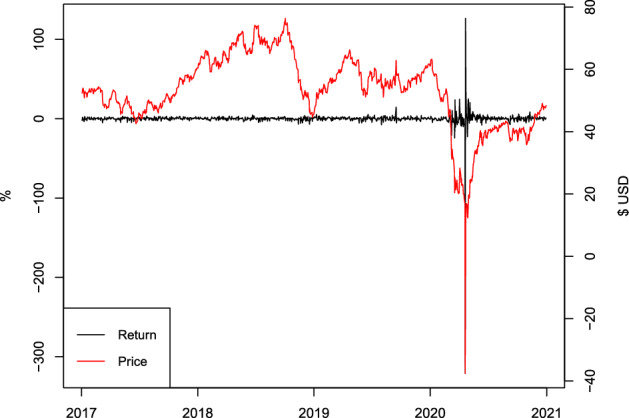
Daily returns and prices of the WTI future: 2017–2020. *Note* This figure plots the daily close prices and returns of WTI future over 2017–2020

On April 20, 2020, the price of West Texas Intermediate (WTI) crude oil future drops by over 300%, trading at $$-\$37.63$$ USD by the end of day (Shi, 2021). This history-writing event, however, does not last long, and the close price of the next trading day increases by over 100% and ends at $10.01 USD. As plotted on Fig. 1, it can be seen that the impacts of those extreme market shocks immediately die out. Volatilities of WTI returns of days following April 21, 2020 seem to recover quickly to a more normal level. Despite this, those extreme shocks can be deemed outliers leading to abnormal volatilities. They may have non-negligible influences when standard statistical models are employed.

Among the existing literature, robust techniques are particularly useful to handle uncertain data exhibiting abnormal observations, as those illustrated in Fig. 1. Under such scenarios, robust techniques are often more efficient and come with more reliable estimates. In particular, advanced robust models, optimization and estimation techniques have been developed and widely employed in operational practices (see, for example, Hladík 2016; Tanveer et al. 2016; Hladík 2019; Černỳ et al. 2020, among others).

This paper examines the performances of robust estimators under the vector autoregressive (VAR) framework, when outliers are unavoidable, especially during the current volatile period. As a standard approach to study the behaviour of multivariate time series, the VAR model has been commonly employed in economics and finance research (Sims, 1980). Rather than a simple extension of the univariate AR model, the popularity of VAR can be attributed to its unique features. For instance, estimates of a VAR model are used to construct metrics known as the impulse response function (IRF) and forecast error variance decomposition (FEVD). Both IRF and FEVD are informative measures to study interrelationships of multivariate economic and financial series (Tsay, 2005).

Broadly speaking, there are two types of outliers in time series (Fox, 1972). Under the VAR framework, an observation is said to be an additive outlier if only its own value has been affected by contamination. In contrast, an observation is treated as an innovational outlier if its corresponding error process is contaminated. Different from an additive outlier, an innovational outlier can affect both the current observation and the subsequent ones. In this paper, we focus on the additive outliers, the impact of which quickly dies out as observed in Fig. 1. Although such outliers have an isolated effect on time series, they can still negatively impact the usual (non-robust) ordinary least squares (OLS) estimation of a VAR model. Further, the accuracy of estimated IRF and FEVD based on the OLS method may also be negatively influenced, causing inappropriate inferences.

Under the scenario of univariate time series, many robust methods have been proposed to handle the potential outliers (Bustos & Yohai, 1986; Pankratz, 1993; McCulloch & Tsay, 1994; Maronna et al., 2006). However, as discussed in Croux and Joossens (2008), compared to univariate time series, dealing with outliers in multivariate time series is more challenging because contamination in one series may be caused by an outlier in other sequences. Among the literature considering robust estimation of multivariate time series models, Ben et al. (1999) extend the Residual Autocovariance (RA)-estimators for univariate ARMA models proposed by Bustos and Yohai (1986) to robustly estimate vector ARMA models. Ben et al. (2006) propose estimating the VAR model based on a $\tau$-scale. Croux and Joossens (2008) utilize a multivariate least trimmed squares estimator (MLTS) as discussed in Agulló et al. (2008) to estimate the VAR model. Because the MLTS estimator usually has a low efficiency, Croux and Joossens (2008) further suggest estimating the VAR model using a reweighted multivariate least trimmed squares (RMLTS) approach to improve the efficiency of the MLTS estimator. The RMLTS estimators can be both robust and efficient. However, choosing the trimming proportions for the RMLTS estimators is relatively ad-hoc. More importantly, the computational cost of MLTS/RMLTS can increase dramatically when the number of observations rises because the optimization problem of MLTS/RMLTS is binary. Therefore, in this paper, we also consider employing a multivariate MM-estimation. The MM-estimation is a two-stage procedure first introduced by Yohai (1987). In the first stage, an M-estimation of the errors scale is calculated based on the initial estimate with high breakdown point (the first M). In the second stage, an M-estimate of regression coefficients is computed based on the scale estimate obtained in the first stage (the second M). Briefly speaking, the MM-estimator is computed as an M-estimator starting at the coefficients provided by a high breakdown S-estimator. Kudraszow and Maronna (2011) further extended the MM-estimator to a multivariate case. As an alternative of RMLTS, MM-estimation is computationally efficient and can achieve both high breakdown point and high efficiency.

Denote the VAR model estimated using the RMLTS and multivariate MM-estimation as RVAR.L and RVAR.M, respectively, this paper aims to demonstrate their effectiveness in the presence of outliers. First, we conduct a comprehensive simulation study for bivariate VAR processes. Altogether, eight scenarios are investigated, allowing for combinations of different true parameters, sample sizes and imposing outliers or not. When outliers are created, compared to the original VAR model (estimated by OLS), RVAR.L and RVAR.M consistently produce more accurate and efficient estimates of the model parameters, as well as those of IRF and FEVD. We also find that the robust VAR models lead to rather similar point estimates and efficiency to their ordinary counterparts when no outliers exist. Nevertheless, both RVAR.L and RVAR.M models demonstrate the desirable asymptotic features, with potentially equivalent asymptotic efficiencies.

Our empirical results include daily realized volatilities with logarithm transformation for six safe haven assets. Those include futures of gold (XAU), silver (XAG), Brent oil (BRE) and WTI oil, and currencies of CHF and JPY, all measured in US dollars. The sample period is July 2017–June 2020, covering the large outliers visualized in Fig. 1. We find that the original VAR model is likely to overestimate the variance of WTI innovation, and various IRFs and FEVDs corresponding to the orthogonal WTI shocks, compared to the robust VAR models. To further explore the validity of robust estimates, the sample is divided into two sub-periods: July 2017–February 2020 (without outliers) and March 2020–June 2020 (with outliers). We find that no significant structural changes are evidenced in the two subsamples for IRF and FEVD. Also, the original and robust VAR models produce rather consistent IRF and FEVD estimates when the first subsample is examined, and when outliers in the second period are removed. This supports the appropriateness of robust estimates obtained using the full sample.

The contributions of this paper are threefold. First, to the best of our knowledge, this is the first study to comprehensively investigate the robust VAR model using the RMLTS and MM multivariate estimators. To accommodate the empirical application of the VAR methodology, we also develop robust counterparties of the popular Akaike information criterion (AIC), the Bayesian information criterion (BIC), and the Hannan–Quinn criterion (HQC) to choose the appropriate number of lags. Second, our simulation study demonstrates the non-negligible biasness of VAR estimated via OLS in the presence of outliers, and more importantly, the effectiveness of RVAR.L and RVAR.M to resolve this issue. This is critical for traders and investors to making trading or investing decisions in practice, especially for the contemporary volatile markets. Finally, our empirical analyses provide important and useful implications for the six commonly employed safety haven assets. Specifically, non-robust estimates could lead to significantly overestimated IRF and FEVD of oil futures corresponding to shocks of XAU, based on which a wrong risk mitigation decision might be made. Also, compared to currencies, the uncertainty of oil futures is less responsive to the orthogonal XAU shocks. Thus, to hedge idiosyncratic risks of the dominant safe haven asset of XAU, one might consider increasing the allocations of oil futures in the portfolio. Those findings also imply that the robust VAR models can be effective tools to study multivariate time series in other operational practices.

The remainder of this paper is organized as follows. Section 2 reviews the ordinary VAR model, and describe the RVAR.L and RVAR.M specifications. Simulation studies are conducted in Sect. 3, and the empirical analyses are presented in Sect. 4. Finally, Sect. 5 concludes.

## Methods

### The vector autoregressive model

Define $\varvec{y}_{t}=(y_{1,t},\ldots ,y_{N,t})'$, an $N\times 1$ vector of time series variables observed at time *t* for $t=1,\ldots , T$, a *p*-th order vector autoregressive model, denoted by VAR(*p*), can be written as:

1 $$\begin{aligned} \varvec{y}_t=\varvec{\mu }+\varvec{A}_1 \varvec{y}_{t-1}+\varvec{A}_2\varvec{y}_{t-2}+ \cdots +\varvec{A}_p\varvec{y}_{t-p}+\varvec{\epsilon }_t, \quad t=1,\ldots , T, \end{aligned}$$

where $\varvec{\mu }$ is an *N*-dimensional intercept vector, $\varvec{A}_i$ for $i=1,\ldots , p$ are $N\times N$ time-invariant coefficient matrices that evaluate the long-run co-movement between time series variables at time *t* and those at time $t-i$, and $\varvec{\epsilon }_t$ is an $N\times 1$ zero-mean white noise vector process (serially independent) with a time-invariant variance-covariance matrix $\varvec{\varSigma }$.

For a covariance stationary VAR system, a range of popular metrics may be employed to perform further analyses. One sufficient condition to ensure the covariance stationarity is the stability, such that the roots of

$$\begin{aligned} \det (\varvec{I}_N - \varvec{A}_1 z - \cdots - \varvec{A}_p z^p ) = 0 \end{aligned}$$

lie outside the complex unit circle, where $\varvec{I}_N$ is the $N \times N$ identity matrix. If this condition is met, $(\varvec{I}_N - \varvec{A}_1 - \cdots - \varvec{A}_p)^{-1}\varvec{\mu }$ then measures the unconditional or long-term mean of $\varvec{y}_t$.

Another popular metric of the VAR analysis is the impulse response function (IRF). For a covariance stationary system, (1) can be written as a moving-average (MA) model, such that

2 $$\begin{aligned} \varvec{y}_t=\varvec{c}+\varvec{\epsilon }_{t}+\varvec{\varPhi }_1\varvec{\epsilon }_{t-1}+\varvec{\varPhi }_2\varvec{\epsilon }_{t-2}+\cdots \end{aligned}$$

Following Lütkepohl (199), it can be shown that

$$\begin{aligned} \varvec{\varPhi }_i = \sum _{j=1}^{i}\varvec{\varPhi }_{i-j}A_j \end{aligned}$$

for $i=1,2,\ldots$, where ${\varPhi }_0$ is an $N \times N$ identity matrix, and $\varvec{A}_j=\varvec{0}$ for $j>p$. The elements of $\varvec{\varPhi }_i$ may be intuitively interpreted as the impulse response. However, due to the non-zero cross-sectional correlations among the error sequence, elements of $\varvec{\varPhi }_s$ for $s>0$ cannot be easily interpreted. To resolve this issue, one may work on the orthogonal errors $\varvec{\eta }_t=\varvec{L}^{-1}\varvec{\epsilon }_{t}$, where $\varvec{L}$ is an invertible lower triangular matrix derived using the Cholesky decomposition as follows

3 $$\begin{aligned} \varvec{\varSigma }=\varvec{L D L}^\prime . \end{aligned}$$

The MA representation (2) can then be rewritten with respect to $\varvec{\eta }_t$:

$$\begin{aligned} \varvec{y}_t=\varvec{c}+\varvec{\varTheta }_1\varvec{\eta }_{t-1}+\varvec{\varTheta }_2\varvec{\eta }_{t-2}+\cdots \end{aligned}$$

IRFs are therefore defined as impulse responses of the *i*th series to the *j*th orthogonal shocks $\eta _{j,t}$, or

$$\begin{aligned} \dfrac{\partial y_{i,t+s}}{\partial \eta _{j,t}}=\dfrac{\partial y_{i,t}}{\partial \eta _{j,t-s}}=\theta ^s_{i,j},\quad i,j=1,\ldots ,N;\;s>0 \end{aligned}$$

where $\theta ^s_{i,j}$ is the *ij*th element of $\varvec{\varTheta }_s$.

Another metric discussed in this paper is the forecast error variance decomposition (FEVD), which is closely related to IRF. In short, the *ij*th *h*-step-ahead FEVD describes the portion of variance of forecast errors in predicting $y_{i,T+h}$ explained by the *j*th orthogonal shocks $\eta _{j,t}$. The mathematical definition is provided below.

4 $$\begin{aligned} \text {FEVD}_{i,j}(h)=\dfrac{\sigma _{\eta _j}^2\sum _{s=0}^{h-1}(\theta _{i,j}^s)^2}{\sum _{j=1}^{N}\sigma _{\eta _j}^2\sum _{s=0}^{h-1}(\theta _{i,j}^s)^2}, \end{aligned}$$

where $\sigma _{\eta _j}^2$ is the variance of $\eta _{j,t}$ and equal to the *j*th diagonal element of $\varvec{D}$ as defined in (3).

In terms of the estimation, assume there are no restrictions put on the parameters $\varvec{A}_i$ for $i=1,\ldots , p$, the VAR(*p*) as in (1) can be rewritten as

$$\begin{aligned} \varvec{y}_t=\varvec{B}'\varvec{z}_{t}+\varvec{\epsilon }_t, \quad t=1,\ldots , T, \end{aligned}$$

where $\varvec{z}_{t}=(1, \varvec{y}'_{t-1}, \ldots , \varvec{y}'_{t-p})'$ is an $(Np+1)\times 1$ vector and $\varvec{B}=(\varvec{\mu }, \varvec{A}_1, \ldots , \varvec{A}_p)'$ is an $(Np+1)\times N$ matrix that contains all unknown coefficients. Alternatively, if we define $\varvec{Y}=(\varvec{y}_1,\ldots ,\varvec{y}_T)'$, $\varvec{Z}=(\varvec{z}_1,\ldots ,\varvec{z}_T)'$, and $\varvec{E}=(\varvec{\epsilon }_1,\ldots ,\varvec{\epsilon }_T)'$, the VAR(*p*) can be represented as a concise matrix form:

$$\begin{aligned} \varvec{Y}=\varvec{Z}\varvec{B}+\varvec{E}. \end{aligned}$$

Therefore, the VAR(*p*) can be estimated using ordinary least squares (OLS) under a multivariate regression framework, which is given by the well-known formula:

5 $$\begin{aligned} \widehat{\varvec{B}}_{\text {OLS}}=(\varvec{Z}'\varvec{Z})^{-1} \varvec{Z}'\varvec{Y}. \end{aligned}$$

Correspondingly, the covariance matrix of the error can be estimated by the sample covariance of the multivariate least square residuals such that:

$$\begin{aligned} \widehat{\varvec{\varSigma }}_{\text {OLS}}=\frac{1}{T-p} \left( \varvec{Y}-\varvec{Z}\widehat{\varvec{B}}_{\text {OLS}} \right) '\left( \varvec{Y}-\varvec{Z} \widehat{\varvec{B}}_{\text {OLS}}\right) . \end{aligned}$$

#### Remark 1

Unfortunately, it is widely known that the OLS estimators for $\varvec{B}$ and $\varvec{\varSigma }$ are very sensitive to outliers since they have a 0 breakdown point. The breakdown point of an estimator is defined as the proportion of ‘bad’ data (i.e., arbitrarily large values) that an estimator can handle without making the estimator arbitrarily bad. Thus, when outliers are present, the OLS estimators often perform poorly, which negatively influences the interpretation and further inferences of an fitted VAR, such as IRF and FEVD.

#### Remark 2

In this study, we consider the Tukey–Huber contamination model (Huber, 1964; Tukey, 1962) that assumes a certain percentage of the data may be affected by abnormal noise. To be more specific, a large proportion (e.g. $1-\varepsilon$, where $\varepsilon$ is usually from 0 to 25%) of the data come from a fully described normal-error model, while the remaining $\varepsilon$ percent of the data can be affected by abnormal fluctuations that arise from the data contamination. Detailed discussions on the Tukey–Huber model can be found in Alqallaf et al. (2009).

### Robust VAR using the multivariate least trimmed squares

To overcome outliers present in multivariate time series, Croux and Joossens (2008) propose to estimate the VAR model by a multivariate least trimmed squares (MLTS) estimator as introduced in Agulló et al. (2008). The MLTS comes from the idea of Minimum Covariance Determinant (MCD) estimator (Rousseeuw & Driessen, 1999) that chooses the subset of *s* observation from *T* observations for which the determinant of the covariance matrix of least-squares residuals is minimal. Specifically, we define ${\mathcal {S}}=\{S\in \{1,\ldots , T\}, |S|=s\}$ be the collection of all possible subsets of size *s*, where *S* is any subset such that $S\in \mathcal {S}$, and let $\widehat{\varvec{B}}_{\text {OLS}}^{(S)}$ be the OLS estimator as in (5) but only using the observations in the subset *S*, such that

$$\begin{aligned} \widehat{\varvec{B}}_{\text {OLS}}^{(S)}=(\varvec{Z}^{(S)'} \varvec{Z}^{(S)})^{-1}\varvec{Z}^{(S)'}\varvec{Y}^{(S)}, \end{aligned}$$

where $\varvec{Z}^{(S)}$ and $\varvec{Y}^{(S)}$ consist of the rows of $\varvec{Z}$ and $\varvec{Y}$ that belong to the subset *S*. Correspondingly, the covariance matrix estimated using this subset *S* is computed as:

$$\begin{aligned} \widehat{\varvec{\varSigma }}_{\text {OLS}}^{(S)}=\frac{1}{T-p} \left( \varvec{Y}^{(S)}-\varvec{Z}^{(S)} \widehat{\varvec{B}}^{(S)}_{\text {OLS}}\right) '\left( \varvec{Y}^{(S)}- \varvec{Z}^{(S)}\widehat{\varvec{B}}^{(S)}_{\text {OLS}}\right) . \end{aligned}$$

Then, the MLTS is defined as:

6 $$\begin{aligned} \widehat{\varvec{B}}_{\text {MLTS}}=\widehat{\varvec{B}}_{\text {OLS}}^{(\widehat{S})}, \quad \text {where}\quad {\widehat{S}}=\mathop {\text {argmin}}\limits _{S\in \mathcal {S}}|\widehat{\varvec{\varSigma }}_{\text {OLS}}^{(S)}|, \end{aligned}$$

where $|\cdot |$ indicates the determinant of a matrix. The associated MLTS estimator of the error covariance matrix is

$$\begin{aligned} \widehat{\varvec{\varSigma }}_{\text {MLTS}}=c_{\alpha } \widehat{\varvec{\varSigma }}_{\text {OLS}}^{(\widehat{S})}, \end{aligned}$$

where $c_{\alpha }$ is a correction factor that ensures the consistency of $\widehat{\varvec{\varSigma }}_{\text {MLTS}}$, and $\alpha$ is known as the trimming proportion for the MLTS estimator such as $\alpha \approx 1-\frac{s}{T}$. The value of $c_{\alpha }$ depends on the error distribution and its detailed calculation under a multivariate normal case is provided in Agulló et al. (2008).

It is worth mentioning that Agulló et al. (2008) also prove that solving the problem of (6) is equivalent to minimizing the sum of the *s* smallest squared Mahalanobis distances of its residuals. That is, we can also define $\widehat{\varvec{B}}_{\text {MLTS}}$ as:

$$\begin{aligned} \widehat{\varvec{B}}_{\text {MLTS}}=\mathop {\text {argmin}}\limits _{\varvec{B}, \varvec{\varSigma }; |\varSigma | =1}\sum _{n=1}^{s} d^2_{(n)}(\varvec{B}, \varvec{\varSigma }), \end{aligned}$$

where $d_{(1)}(\varvec{B}, \varvec{\varSigma })\le d_{(2)}(\varvec{B}, \varvec{\varSigma })\ldots \le d_{(n)}(\varvec{B}, \varvec{\varSigma })$ is the ordered sequence of the residual Mahalanobis distance, which is given by:

7 $$\begin{aligned} d_{t}(\varvec{B}, \varvec{\varSigma })=\left[ (\varvec{y}_t-\varvec{B}'\varvec{z}_{t})' \varvec{\varSigma }^{-1}(\varvec{y}_t-\varvec{B}'\varvec{z}_{t})\right] ^{\frac{1}{2}}. \end{aligned}$$

Therefore, the MLTS estimator can be viewed as a multivariate extension of the Least Trimmed Squares (LTS) estimator of Rousseeuw (1984).

Although the MLTS estimator can be robust against outliers, it usually has a low efficiency. A reweighed step is commonly implemented to improve the efficiency of the MLTS estimator. Following Agulló et al. (2008), the reweighted multivariate least trimmed squares (RMLTS) estimators for $\varvec{B}$ and $\varvec{\varSigma }$ are given by

$$\begin{aligned} \widehat{\varvec{B}}_{\text {RMLTS}}=\widehat{\varvec{B}}_{\text {OLS}}^{(J)}\quad \text {and}\quad \widehat{\varvec{\varSigma }}_{\text {RMLTS}}=c_{\alpha } \widehat{\varvec{\varSigma }}_{\text {OLS}}^{(J)}, \end{aligned}$$

respectively, where $J=\{j\in \{1,\ldots , T\}| d_{j}(\varvec{B}, \varvec{\varSigma })\le \chi ^2_{Np+1,1-\delta }\}$, and $\chi ^2_{Np+1,1-\delta }$ is the upper $\delta$-quantile of a Chi-square distribution with $Np+1$ degrees of freedom. After a model is estimated by MLTS, the residual with a Mahalanobis distance $d_{j}(\widehat{\varvec{B}}_{\text {MLTS}}, \widehat{\varvec{\varSigma }}_{\text {MLTS}})$ larger than the critical value $q_{\delta }$ is then identified as an outlier. RMLTS is then computed using data which are non-outliers. A standard choice of $\alpha$ and $\delta$ is 0.25 and 0.01, respectively, which is also adopted in this study. Agulló et al. (2008) show that when $N=2$, the RMLTS achieves an asymptotic efficiency of 0.941 for the normal distribution, whereas this efficiency of MLTS is only 0.403.

### Robust VAR using multivariate S- and MM-estimation

Despite that RMLTS estimators can achieve a high breakdown point and high efficiency, choosing the trimming proportions $\alpha$ and $\delta$ are relatively ad-hoc. In addition, because the optimization problem of MLTS/RMLTS is binary, finding the best subset $\widehat{S}$ is computationally expensive when the total number of observations *T* rises. Thus, we consider another commonly used robust estimation method, or the S-estimation (Rousseeuw & Yohai, 1984). Van Aelst and Willems (2005) extend the S-estimator to a multivariate case, which can be adopted to estimate the VAR(*p*) model. In the context of the VAR(*p*) model, we consider the S-estimator as:

8 $$\begin{aligned} (\widehat{\varvec{B}}_{\text {S}},\widehat{\varvec{\varSigma }}_{\text {S}})=\mathop {\text {argmin}}\limits _{\varvec{B}, \varvec{\varSigma }} |{\varvec{\varSigma }}|,\text { subject to } \frac{1}{T}\sum _{t=1}^{T}\rho \left( d_{t}(\varvec{B}, \varvec{\varSigma })\right) =b, \end{aligned}$$

where $d_{t}(\varvec{B}, \varvec{\varSigma })$ is the residual Mahalanobis distance for *t*-th observation as defined in (7), and $b=E(\rho (d_{t}(\cdot )))$ is the expected value of $\rho (d_{t}(\cdot ))$ under the error distribution, which is usually set as 0.5 to ensure the maximal asymptotic breakdown point. The loss function $\rho$ is assumed to satisfy the following two properties:

1. $\rho$ is twice continuously differentiable and symmetric at $\rho (0)=0$; and
2. $\rho$ is strictly increasing on [0, *d*] and eventually constant.

A commonly used family of loss functions $\rho$ is given by the Tukey’s biweight,

$$\begin{aligned} \rho (u)=\left\{ \begin{array}{ll} \frac{d^2}{6}\left( 1-\left[ 1- \left( \frac{u}{d}\right) ^2\right] ^3\right) &{}\quad \hbox {if}\; |u|\le d\\ \frac{d^2}{6} &{}\quad \hbox {if}\; |u|\ge d.\end{array}\right. \end{aligned}$$

where *d* is a tuning constant that controls the trade-off between robustness and efficiency, which is usually chosen at the value that achieves a 0.5 breakdown point. The detailed algorithm to solve the multivariate S-estimator is provided in Van Aelst and Willems (2005). Although the multivariate S-estimator is highly robust against outliers, its efficiency is relatively low when the number of time series *N* is not large. According to Van Aelst and Willems (2005), when $N=2$ and the error distribution is normal, the asymptotic efficiency of the multivariate S-estimator is only 0.580.

To resolve this, we explore the MM-estimation instead, which achieves both high breakdown point and high efficiency. The MM-estimation is a two-stage procedure first introduced by Yohai (1987) and further extended to a multivariate case by Kudraszow and Maronna (2011). In the first stage, an initial estimator (e.g. S-estimators for both $\varvec{B}$ and $\varvec{\varSigma }$) is computed with high breakdown point but not necessarily efficient. In the second stage, an M-estimator for $\varvec{B}$ is computed based on the scale estimator (e.g. S-estimator for $\varvec{\varSigma }$) obtained in the first stage. Briefly speaking, an MM-estimator is computed as an M-estimator starting at the coefficients given by a high breakdown S-estimator and with a fixed scale also provided by the S-estimator.

To sum up, in this study, we consider the multivariate MM-estimation of the VAR(*p*) model with the following two steps:

- Step 1: Compute the S-estimators $(\widehat{\varvec{B}}_{\text {S}},\widehat{\varvec{\varSigma }}_{\text {S}})$ according to (8) with a loss function $\rho _1$ and $b=0.5$;
- Step 2: Obtain the MM-estimator $\widehat{\varvec{B}}_{\text {MM}}$ given as
  $$\begin{aligned} \widehat{\varvec{B}}_{\text {MM}}=\mathop {\text {argmin}}\limits _{\varvec{B}}\frac{1}{T}\sum _{t=1}^{T}\rho _2\left( d_{t}(\varvec{B}, \widehat{\varvec{\varSigma }}_{\text {S}})\right) , \end{aligned}$$
  and assign $$\widehat{\varvec{\varSigma }}_{\text {MM}}=\widehat{\varvec{\varSigma }}_{\text {S}}$$,

where two loss functions $\rho _1$ and $\rho _2$ both satisfy properties A1 and A2 and are subject to $\rho _1\ge \rho _2$. If we let $d_1$ and $d_2$ be the tuning constants for $\rho _1$ and $\rho _2$, respectively, then the breakdown point of MM-estimator only depends on $d_1$, such that its desired efficiency can be achieved by choosing an appropriate value of $d_2$ but without affecting the breakdown point.

#### Remark 3

As discussed above, compared to the MLTS and multivariate S-estimators, the corresponding RMLTS and the multivariate MM-estimators are both robust and more efficient. Thus, we only focus on estimating the VAR(*p*) model using these two robust estimators in our simulation study and empirical analysis.

#### Remark 4

To derive the robust IRF and FEVD as discussed in Sect. 2.1, the same formula are followed. In all cases, estimators of $\varvec{A}_i$ and $\varSigma$ produced by the OLS are replaced by their robust counterparties.

### Lag selection using robust information criteria

Following the above description, the VAR(*p*) model can be robustly estimated using the RMLTS or the multivariate MM-estimation for a given *p*. However, *p* is usually not known in advance and needs to be determined by the data. One standard approach is to fit the VAR(*p*) model with lags $p=1,\ldots , p_{{\textit{max}}}$ and select the optimal value of *p* which minimizes some model selection criteria. Three commonly used information criteria (IC) for the VAR are the Akaike information criterion (AIC), Bayesian information criterion (BIC) and Hannan–Quinn criterion (HQC), all of which are in a form of the negative penalized log-likelihood function given as follows,

9 $$\begin{aligned} {\textit{IC}}(p)=\ln |\widehat{\varvec{\varSigma }}_{\text {OLS}}(p)|+K^2p\cdot \tau (T), \end{aligned}$$

where $\widehat{\varvec{\varSigma }}_{\text {OLS}}(p)$ is the residual covariance matrix using lag *p*, $K^2p$ is the number of parameters, $\tau (T)$ is a function of *T*, and $K^2p\cdot \tau (T)$ serves as a penalty function which penalizes the VAR model with a large number of parameters.

However, the presence of outliers not only affects the estimators, but also (and sometimes more severely) the model selection procedure using the likelihood based criteria as in (9), since $\widehat{\varvec{\varSigma }}_{\text {OLS}}$ is very sensitive to outliers. To select the optimal *p* for robust VAR models, in this work, we propose a robust model selection approach based on a robust version of the penalized log-likelihood.

Consider the log-likelihood of VAR(*p*) under a multivariate normal error distribution:

$$\begin{aligned} l(\varvec{B}, \varvec{\varSigma })=-\frac{T}{2}\ln |\varvec{\varSigma }|-\frac{NT}{2}\ln (2\pi )-\frac{1}{2} \sum _{t=1}^{T}d^2_{t}(\varvec{B}, \varvec{\varSigma }). \end{aligned}$$

When $(\varvec{B}, \varvec{\varSigma })$ are estimated by the OLS, we observe that

$$\begin{aligned} \sum _{t=1}^{T}d^2_{t}(\widehat{\varvec{B}}_{\text {OLS}}, \widehat{\varvec{\varSigma }}_{\text {OLS}})=\sum _{t=1}^{T} (\varvec{y}_t-\widehat{\varvec{B}}_{\text {OLS}}'\varvec{z}_{t})' \widehat{\varvec{\varSigma }}_{\text {OLS}}^{-1} (\varvec{y}_t-\widehat{\varvec{B}}_{\text {OLS}}'\varvec{z}_{t})= (T-N)N, \end{aligned}$$

which becomes a constant, and thus, the negative $l(\widehat{\varvec{B}}_{\text {OLS}},\widehat{\varvec{\varSigma }}_{\text {OLS}})$ coincides with $\ln |\widehat{\varvec{\varSigma }}_{\text {OLS}}|$ by dropping out the constant term. However, when $(\varvec{B}, \varvec{\varSigma })$ are estimated by robust methods (e.g. the RMLTS or the multivariate MM-estimation), which we denote by $(\widetilde{\varvec{B}},\widetilde{\varvec{\varSigma }})$, $\sum _{t=1}^{T}d^2_{t}(\widetilde{\varvec{B}},\widetilde{\varvec{\varSigma }})$ is no longer a constant term and it will be dominated by potential outliers with large residual Mahalanobis distances. Therefore, in the same spirit of robust estimation on the model parameters, we propose the following robust information criterion (RIC) to bound the influence of potential outliers that have large residual Mahalanobis distances:

10 $$\begin{aligned} {\textit{RIC}}(p)=\frac{1}{T}\sum _{t=1}^{T}\tilde{\rho }\left( d_{t}(\widetilde{\varvec{B}}(p),\widetilde{\varvec{\varSigma }}(p))\right) +\ln |\widetilde{\varvec{\varSigma }}(p)|+K^2p\cdot \tau (T). \end{aligned}$$

As discussed in Müller and Welsh (2005), linking the information criterion to any estimators (e.g. use the same $\rho$ function in the selection criterion and estimation) may excessively favor that estimator. As a result, in this study, we choose $\tilde{\rho }$ in (10) by Huber’s loss, which is not used in either the RMLTS or the multivariate MM-estimation:

$$\begin{aligned} \tilde{\rho }(u)=\left\{ \begin{array}{ll} {u^2}&{}\quad \hbox {if}\; |u|\le c\\ 2c|u|-{c^2} &{} \quad \text {otherwise},\end{array}\right. \end{aligned}$$

where *c* is a tuning constant that controls the amount of the robustness. In conclusion, we consider robust versions of three aforementioned criteria (AIC, BIC, and HQC) which correspond to RIC with three different types of the penalty function:

$$\begin{aligned} R.{\textit{AIC}}(p)=&\frac{1}{T}\sum _{t=1}^{T}\tilde{\rho }\left( d_{t}(\widetilde{\varvec{B}}(p),\widetilde{\varvec{\varSigma }}(p))\right) +\ln |\widetilde{\varvec{\varSigma }}(p)|+K^2p\cdot \frac{2}{T};\\ R.{\textit{BIC}}(p)=&\frac{1}{T}\sum _{t=1}^{T}\tilde{\rho }\left( d_{t}(\widetilde{\varvec{B}}(p),\widetilde{\varvec{\varSigma }}(p))\right) +\ln |\widetilde{\varvec{\varSigma }}(p)|+K^2p\cdot \frac{\ln (T)}{T};\\ R.{\textit{HQC}}(p)=&\frac{1}{T}\sum _{t=1}^{T}\tilde{\rho }\left( d_{t}(\widetilde{\varvec{B}}(p),\widetilde{\varvec{\varSigma }}(p))\right) +\ln |\widetilde{\varvec{\varSigma }}(p)|+K^2p\cdot \frac{2\ln \ln (T)}{T}. \end{aligned}$$

The optimal *p* for robust VAR models is therefore selected by the one that minimizes one of the above criteria. Owing to its preference on a more parsimonious model, we employ the (robust) BIC throughout the rest of this paper. Our results and conclusions, however, consistently hold when the (robust) AIC or HQC is employed instead.^1^

## Simulation study

In this section, we carry out a simulation study to compare the performances of original and robust VAR models with various simulation settings. Section 3.1 describes those settings. Sections 3.2 and 3.3 discuss the estimation under scenarios with and without outliers, respectively.

### Simulation setting

Throughout this section, we consider a bivariate ($N=2$) VAR model with lag $p=1$ as follows:

11 $$\begin{aligned} \varvec{y}_t=\varvec{\mu }+\varvec{A} \varvec{y}_{t-1}+\varvec{\epsilon }_t, \quad t=1,\ldots , T, \end{aligned}$$

where $\varvec{A}$ is a $2\times 2$ matrix of coefficients, and we assume that the error follows a bivariate normal distribution with mean $\varvec{0}$ and covariance $\varvec{\varSigma }$ (a $2\times 2$ matrix). In our simulation, we let

$$\begin{aligned} \varvec{\mu }=\begin{bmatrix} 0 \\ 0 \end{bmatrix},\quad \varvec{A}=\begin{bmatrix} 0.6 &{}0.3\\ 0.3 &{}0.6 \end{bmatrix}, \quad \varvec{\varSigma }=\begin{bmatrix} 1 &{} r\\ r &{} 1 \end{bmatrix}, \end{aligned}$$

where the error terms for both variables have a variance of 1, and *r* measures the correlation between the bivariate errors.^2^

In order to investigate the performance of robust VAR against outliers, we focus on data generated by (11) but with 1% of the observations being contaminated. That is, once *T* vectors of $\varvec{y}_t$ are simulated, $1\%\times T$ out of *T* randomly selected vectors of $\varvec{y}_t$ have random entries contaminated by numbers generated from *N*(10, 1). To ease the comparison, we assume that the lag $p=1$ is known in advance in our simulation study and consider the following settings with various values of *T* and *r*.

1. $T=100$ (small) and $r=0.2$ (low);
2. $T=500$ (large) and $r=0.2$ (low);
3. $T=100$ (small) and $r=0.8$ (high);
4. $T=500$ (large) and $r=0.8$ (high).

For each setting, we produce 1000 replicates, and simulated samples are fitted by the original VAR, robust VAR with RMLTS estimator (RVAR.L) and robust VAR with MM-estimator (RVAR.M) models individually. The same settings are then employed to construct scenarios without outliers to compare all models.

### Scenarios with outliers

We first consider the scenarios with outliers. The absolute bias and standard errors (SEs) of the residual variance-covariance matrix (${\varvec{\varSigma }}$), which are calculated using the estimates and true values, are reported in Table 1. For its incompatibility with outliers, the original VAR model leads to considerably large bias and SEs in all cases. For instance, for a small sample size with a low correlation in cross-sectional errors, absolute bias (SE) of the diagonal elements of $\varvec{\varSigma }$ is mostly above 0.48 (0.58). When *r* increases to 0.8, the absolute bias and SE are similar to their counterparties when $r=0.2$. In contrast, the absolute biases produced by RVAR.L and RVAR.M models are mostly below 0.05 and only marginally vary with different *r*. With respect to the estimation efficiency, SEs of the robust models are over 50% smaller than those of the original VAR model. Consequently, even with a small sample size of 100, RVAR.L and RVAR.M provide much less biased and more efficient estimates than the original VAR model. Nevertheless, when the sample size increases to 500, the absolute bias of robust models reduces to much negligible levels (only up to 0.025). The improved efficiency of estimation is also evidenced, since SEs are substantially below 0.1. Similarly, also from Table 1, much identical conclusions can be drawn for the estimated auto-regressive matrix $\varvec{A}$. Such results demonstrate the desirable asymptotic consistency and efficiency of RVAR.L and RVAR.M models, when estimating VAR parameters with the existence of large outliers.^3^

**Table 1:** Simulation results of the estimated parameters: With outliers

*r*	*T*	Model	$\sigma _{1}^2$		$\sigma _{12}$		$\sigma _{2}^2$			
			Bias	SE	Bias	SE	Bias	SE		
*Variance-covariance matrix*										
0.2	100	VAR	0.534	0.639	0.120	0.153	0.703	0.610		
		RVAR.L	0.023	0.207	0.020	0.144	0.055	0.181		
		RVAR.M	0.005	0.167	0.021	0.116	0.027	0.149		
	500	VAR	0.747	0.294	0.147	0.074	0.630	0.304		
		RVAR.L	0.002	0.087	0.008	0.067	0.008	0.082		
		RVAR.M	0.025	0.068	0.001	0.048	0.023	0.070		
0.8	100	VAR	0.487	0.595	0.062	0.164	0.647	0.585		
		RVAR.L	0.012	0.204	0.005	0.183	0.028	0.196		
		RVAR.M	0.010	0.164	0.008	0.145	0.011	0.157		
	500	VAR	0.723	0.277	0.144	0.092	0.608	0.288		
		RVAR.L	0.008	0.087	0.010	0.079	0.011	0.083		
		RVAR.M	0.020	0.068	0.013	0.061	0.020	0.068		

*r*	*T*	Model	$\beta _{11}$		$\beta _{12}$		$\beta _{21}$		$\beta _{22}$	
			Bias	SE	Bias	SE	Bias	SE	Bias	SE
*Auto-regressive matrix*										
0.2	100	VAR	0.117	0.184	0.016	0.179	0.021	0.176	0.116	0.192
		RVAR.L	0.028	0.121	0.023	0.121	0.004	0.124	0.040	0.122
		RVAR.M	0.025	0.108	0.030	0.110	0.010	0.116	0.054	0.125
	500	VAR	0.089	0.079	0.009	0.068	0.030	0.074	0.107	0.078
		RVAR.L	0.008	0.035	0.005	0.041	0.007	0.042	0.016	0.040
		RVAR.M	0.008	0.033	0.004	0.038	0.007	0.039	0.016	0.038
0.8	100	VAR	0.156	0.324	0.096	0.327	0.082	0.322	0.143	0.330
		RVAR.L	0.023	0.259	0.024	0.266	0.025	0.257	0.063	0.263
		RVAR.M	0.044	0.245	0.005	0.256	0.014	0.241	0.059	0.253
	500	VAR	0.119	0.140	0.064	0.133	0.097	0.139	0.150	0.139
		RVAR.L	0.000	0.068	0.011	0.073	0.013	0.075	0.023	0.075
		RVAR.M	0.003	0.078	0.015	0.081	0.025	0.094	0.035	0.097

This table presents simulation results of the estimated parameters when outliers are created in each replicate. The models compared are the original VAR, robust VAR using the reweighted multivariate least trimmed squares estimator (RVAR.L) and robust VAR using the MM-estimator (RVAR.M). *r* is the correlation between the innovations of the two series. *T* is the sample size. Bias is the absolute bias. SE is the standard error. The number of replicates is 1000 for each simulation setting

**Table 2:** Simulation results of essential VAR statistics: with outliers

*r*	*T*	Model	IRF				FEVD	
			1-to-1	1-to-2	2-to-1	2-to-2	1-in-1	2-in-2
0.2	100	VAR	0.163	0.137	0.126	0.151	0.087	0.099
		RVAR.L	0.111	0.117	0.101	0.104	0.082	0.119
		RVAR.M	0.099	0.104	0.099	0.102	0.078	0.098
	500	VAR	0.126	0.098	0.078	0.118	0.044	0.052
		RVAR.L	0.050	0.055	0.042	0.044	0.033	0.052
		RVAR.M	0.043	0.045	0.038	0.040	0.030	0.041
0.8	100	VAR	0.195	0.177	0.158	0.202	0.108	0.279
		RVAR.L	0.144	0.145	0.103	0.106	0.049	0.082
		RVAR.M	0.132	0.134	0.098	0.099	0.050	0.070
	500	VAR	0.140	0.108	0.097	0.166	0.062	0.225
		RVAR.L	0.066	0.067	0.037	0.039	0.018	0.035
		RVAR.M	0.058	0.058	0.039	0.041	0.019	0.030

This table presents the root of mean squared errors (RMSE) of the long-term mean, impulse response functions (IRFs) and forecast error variance decomposition (FEVD) when 1% large outlier is allowed in each replicate. The models compared are the original VAR, RVAR.L and RVAR.M. *1-to-1* and *2-to-1* (*1-to-2* and *2-to-2*) are RMSEs of IRFs for $y_1$ and $y_2$ to changes in $y_1$ ($y_2$), respectively. *1-in-1* (*2-in-2*) is the RMSE of FEVD for $y_1$ ($y_2$) in explaining the forecast errors of $y_1$ ($y_2$). Note that both IRFs and FEVD are produced to 10 steps, and RMSEs reported in the table are averages over the 10 steps

We now discuss the two common metrics to perform further analyses of a VAR system: IRF and FEVD. In this case, we focus on the root of mean squared error (RMSE) as an overall estimation accuracy measure. RMSE is constructed as the root of summation of squared bias and SE, and the corresponding results are reported in Table 2. For both IRF and FEVD, we consider up to 10 steps, and the average of RMSEs over all the 10 steps is presented. Besides, from (4), it is also worth noting that FEVDs of the *i*th series always sum up to 100%. Consequently, we only focus on FEVD that is explained by the series itself. In a binary case, large (or small) RMSEs of FEVD are most likely to occur simultaneously when investigating the same sequence.

**Table 3:** Simulation results of the estimated parameters: no outliers

*r*	*T*	Model	$\sigma _{1}^2$		$\sigma _{12}$		$\sigma _{2}^2$			
			Bias	SE	Bias	SE	Bias	SE		
*Variance-covariance matrix*										
0.2	100	VAR	0.004	0.149	0.013	0.110	0.030	0.128		
		RVAR.L	0.043	0.208	0.016	0.148	0.063	0.180		
		RVAR.M	0.036	0.159	0.009	0.110	0.066	0.141		
	500	VAR	0.004	0.058	0.008	0.044	0.001	0.066		
		RVAR.L	0.016	0.090	0.010	0.067	0.020	0.082		
		RVAR.M	0.015	0.064	0.010	0.045	0.015	0.069		
0.8	100	VAR	0.007	0.145	0.003	0.131	0.014	0.136		
		RVAR.L	0.030	0.210	0.019	0.187	0.043	0.194		
		RVAR.M	0.030	0.158	0.024	0.140	0.048	0.149		
	500	VAR	0.008	0.059	0.008	0.055	0.005	0.063		
		RVAR.L	0.021	0.087	0.020	0.077	0.023	0.081		
		RVAR.M	0.019	0.064	0.018	0.058	0.019	0.066		

*r*	*T*	Model	$\beta _{11}$		$\beta _{12}$		$\beta _{21}$		$\beta _{22}$	
			Bias	SE	Bias	SE	Bias	SE	Bias	SE
*Auto-regressive matrix*										
0.2	100	VAR	0.025	0.088	0.023	0.100	0.002	0.096	0.036	0.092
		RVAR.L	0.025	0.117	0.029	0.117	0.004	0.116	0.036	0.108
		RVAR.M	0.025	0.094	0.024	0.103	0.000	0.097	0.035	0.091
	500	VAR	0.006	0.033	0.005	0.037	0.008	0.038	0.017	0.037
		RVAR.L	0.007	0.036	0.005	0.040	0.008	0.042	0.017	0.041
		RVAR.M	0.007	0.033	0.005	0.037	0.008	0.039	0.016	0.037
0.8	100	VAR	0.012	0.175	0.034	0.187	0.008	0.182	0.049	0.186
		RVAR.L	0.006	0.221	0.041	0.224	0.016	0.221	0.055	0.220
		RVAR.M	0.011	0.181	0.035	0.192	0.010	0.183	0.050	0.187
	500	VAR	0.003	0.066	0.013	0.069	0.016	0.071	0.026	0.070
		RVAR.L	0.001	0.068	0.013	0.072	0.015	0.074	0.025	0.074
		RVAR.M	0.002	0.066	0.013	0.068	0.015	0.070	0.025	0.070

This table presents simulation results of the estimated parameters when no outliers are allowed. The models compared are the original VAR, RVAR.L and RVAR.M

**Table 4:** Simulation results of essential VAR statistics: no outliers

*r*	*T*	Model	IRF				FEVD	
			1-to-1	1-to-2	2-to-1	2-to-2	1-in-1	2-in-2
0.2	100	VAR	0.095	0.098	0.089	0.090	0.074	0.091
		RVAR.L	0.113	0.118	0.104	0.104	0.083	0.121
		RVAR.M	0.098	0.099	0.093	0.094	0.074	0.090
	500	VAR	0.041	0.043	0.035	0.037	0.028	0.040
		RVAR.L	0.051	0.054	0.041	0.045	0.033	0.052
		RVAR.M	0.044	0.045	0.038	0.040	0.029	0.041
0.8	100	VAR	0.126	0.126	0.081	0.083	0.036	0.061
		RVAR.L	0.146	0.145	0.093	0.096	0.038	0.076
		RVAR.M	0.131	0.131	0.082	0.084	0.035	0.059
	500	VAR	0.054	0.055	0.033	0.035	0.017	0.028
		RVAR.L	0.067	0.068	0.036	0.039	0.018	0.034
		RVAR.M	0.059	0.059	0.034	0.036	0.017	0.028

This table presents the RMSE of IRFs and FEVD when no outliers are allowed in each replicate. The models compared are the original VAR, RVAR.L and RVAR.M

Recall that both IRF and FEVD are related to the estimated residual variance-covariance matrix. The inaccuracy in estimating any of those parameters can therefore result in imprecision of those metrics. When both the sample size and cross-sectional correlation are small, robust estimates of IRFs are uniformly more accurate than those of the original VAR model. Although the difference in FEVD is relatively small in this case, the average RMSEs of robust VAR models are over 50% smaller than those of VAR, when $r=0.8$. Finally, with a moderately large sample size of 500, we observe that RVAR.L and RVAR.M consistently outperform VAR in estimating IRFs and FEVDs with considerably distinct improvements.

To sum up, the robust VAR models investigated in this paper provide much more accurate estimates than the original VAR model, when outliers are present. This conclusion holds for both the model parameters, and commonly used metrics including IRF and FEVD. Also, robust VAR models demonstrate desirable asymptotic consistency and efficiency, when the sample size increases. Nevertheless, the performances of RVAR.L and RVAR.M are fairly similar, such that no estimator overall dominates the other.

### Scenarios without outliers

We now investigate the scenarios without large outliers that are manually created. The original VAR model is expected to be adequate in such cases, and it is of interest to explore the relative performance of the robust counterparties.

The results of $\varvec{\varSigma }$ are summarized in Table 3. Even for a small sample size, the absolute bias is reasonably small and close to 0 across all models. It can be concluded from SEs that the estimation efficiency only marginally differs across small and large cross-sectional correlations in errors. Further, all models demonstrate desirable asymptotic behaviour, as both absolute bias and SE consistently reduce when *T* increases to 500. Comparing these three models, the original VAR (RVAR.L) model produces the smallest (largest) SE when $T=100$. Despite this, such differences are much indistinguishable when $T=500$. Also from Table 3, much identical conclusions can be drawn for the estimated auto-regressive matrix $\varvec{A}$.

In Table 4, we present RMSEs of IRF and FEVD of these three models when no outliers exist in the simulated data. Our observation is consistent with that of the residual covariance matrix. Therefore, when no outliers are present, we conclude that for both the model parameters and related metrics, robust VAR models produce much similar results to the original VAR (the true model). It is worth noting that for a small sample size, RVAR.L may be relatively less efficient, compared to the RVAR.M model when estimating VAR parameters, IRF and FEVD. The difference, however, is almost indistinguishable for a moderately large sample size. This indicates the asymptotic equivalence in the efficiency for RMLTS and multivariate MM-estimators.

## Empirical analysis

In this section, we compare the robust and original VAR models using daily realized volatilities of six safe haven asset prices over July 2017–June 2020. The investigated assets are futures of gold (XAU), silver (XAG), Brent oil (BRE) and West Texas Intermediate oil (WTI) and currencies of Swiss Francs (CHF) and Japanese Yen (JPY). All prices are measured in the US dollars. All empirical data are sourced from the Thomson Reuters Tick History. The daily volatility is calculate as the root of summation of squared hourly close prices. Section 4.1 provides an overview of the data, and Sect. 4.2 presents a comprehensive analysis using the original and robust VAR models.

**Fig. 2:**
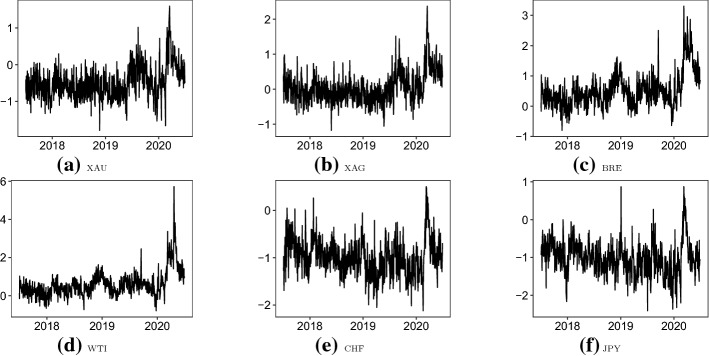
Daily logged volatilities: July 2017–June 2020. *Note* This figure plots the daily logged volatilities of spot prices of WTI oil (WTI), Brent oil (BRE), gold (XAU), silver (XAG), exchange rates of USD/CHF (CHF) and exchange rate of USD/JPY (JPY) over July 2017–June 2020. The volatilities are calculated as realized volatilities using the hourly close prices

### An overview of the data

The logged daily volatilities over the full sample period are presented in Fig. 2. Despite differences in scales, it can be seen that all series are at stable levels until early-2020. Due to the impact of the recent COVID-19 pandemic, all the six assets demonstrate growing volatilities in roughly March 2020. The high levels of uncertainty do not last long, and volatilities return to more normal levels by the end of June 2020. In terms of the abnormal observations, the historical-writing negative price of WTI does lead to large outliers over April 20–22, 2020. For instance, after taking logs, the largest volatility (at around 6) is still almost 10 times larger than the sample mean for WTI (at around 0.59). Thus, as evidenced in Sect. 3, the original VAR model may produce inaccurate estimates when employed to make inferences.

Descriptive statistics of the investigated data are presented in Table 5. In addition to the full sample period, we consider July 2017–February 2020 (without outliers) and March 2020–June 2020 (with outliers). For the full period, it can be seen that currencies are the least volatile sequences, with the smallest level of uncertainty in volatilities, as measured by the standard deviation. Oils, however, are the most volatile assets with the largest level of uncertainty in volatilities. Due to the existence of outliers in end-April 2020, many sample descriptive statistics exhibit unusual values. The skewness and kurtosis of WTI, for instance, largely deviate from 0 and 3, respectively. Much consistent conclusions are drawn, when the period of March 2020–June 2020 is investigated, despite differences caused by sample sizes. In comparison, when the period without outliers is considered, uncertainty measured by the standard deviation is at similar levels for the six assets. Skewness is not too far from 0, and kurtosis is reasonably close to 3.

**Table 5:** Descriptive statistics

	Mean	Std. Dev.	Median	Q$_1$	Q$_3$	Skew.	Kurt.
*Panel A: July 2017–June 2020*							
XAU	$-$0.485	0.445	$-$0.534	$-$0.783	$-$0.267	0.847	4.657
XAG	0.046	0.441	$-$0.009	$-$0.257	0.251	1.046	5.322
BRE	0.525	0.560	0.435	0.167	0.756	1.286	5.617
WTI	0.595	0.671	0.491	0.190	0.811	2.145	11.890
CHF	$-$0.993	0.375	$-$1.014	$-$1.231	$-$0.773	0.472	3.848
JPY	$-$1.018	0.442	$-$1.022	$-$1.301	$-$0.761	0.388	4.475
*Panel B: July 2017–February 2020*							
XAU	$-$0.558	0.382	$-$0.593	$-$0.820	$-$0.342	0.526	3.968
XAG	$-$0.039	0.355	$-$0.058	$-$0.279	0.162	0.570	4.074
BRE	0.391	0.391	0.389	0.127	0.607	0.466	4.240
WTI	0.430	0.409	0.428	0.157	0.679	0.271	3.819
CHF	$-$1.031	0.346	$-$1.033	$-$1.253	$-$0.816	0.201	3.307
JPY	$-$1.054	0.410	$-$1.041	$-$1.325	$-$0.780	$-$0.036	3.745
*Panel C: March 2020–June 2020*							
XAU	0.108	0.474	0.047	$-$0.251	0.265	0.944	3.590
XAG	0.739	0.458	0.683	0.392	0.895	1.164	4.517
BRE	1.599	0.559	1.543	1.282	1.883	0.531	3.244
WTI	1.922	0.876	1.680	1.413	2.304	1.626	7.081
CHF	$-$0.686	0.448	$-$0.767	$-$1.037	$-$0.399	0.645	2.894
JPY	$-$0.732	0.575	$-$0.894	$-$1.139	$-$0.384	0.878	3.106

This table presents descriptive statistics of the daily logged volatilities of spot prices of WTI oil (WTI), Brent oil (BRE), gold (XAU), silver (XAG), exchange rates of USD/CHF (CHF) and exchange rate of USD/JPY (JPY) over three periods: July 2017–June 2020, July 2020–February 2020, and March 2020–June 2020. Std. Dev. is the standard deviation. Q$_1$ and Q$_3$ are the first and third quartiles, respectively. Skew. is the skewness. Kurt. is the kurtosis

Based on the above observations, the full sample period is employed as baseline results of this paper. The two sub-periods are used for further analyses. Those include the comparison of original and robust VAR models without outliers and the potential structural changes in VAR metrics. The examined data are fitted by each of the three models individually, with *p* chosen using the (robust) BIC.

### Estimation results

In this section, we discuss and contrast empirical findings of the model parameters ($\widehat{\varvec{\varSigma }}$), IRF and FEVD over different sample periods and across the original and robust VAR models. Some implications of our empirical results are further discussed.

#### Model parameters

As discussed in Sect. 2, the presence of outliers can largely impact the accuracy in parameter estimation using the original VAR model. To illustrate this, we present $\widehat{\varvec{\varSigma }}$ in Table 6.^4^

We firstly focus on the full sample. It can be observed that all covariances (off-diagonal elements of $\widehat{\varvec{\varSigma }}$) are positive, suggesting the expected co-movements of those popular safe haven assets. Moreover, consistent in all models, we can infer that the corresponding correlations of pairs of precious metal (at around 0.76), oils (at round 0.85), and currencies (at round 0.6) are larger than cross-assets correlations. Roughly speaking, those between precious metal and currencies, previous metal and oils, and oils and currencies are at round 0.4, 0.2 and 0.15, respectively. Despite the closeness in estimating correlations, the estimated $\widehat{\varvec{\varSigma }}$ differ substantially when contrasting the original and robust VAR models. For instance, according to VAR, the estimated variance of errors in WTI is 0.156, which is roughly 40% larger than 0.111 and 0.115 produced by the RVAR.L and RVAR.M models, respectively. This difference may result in non-negligible deviation when calculating IRF and FEVD that are correspondent to WTI errors.

In contrast, when the normal period of July 2017–February 2020 is examined, only marginal differences are observed between the original and robust VAR models. This is consistent with our observation in Sect. 3.3, such that VAR, RVAR.L and RVAR.M produce much identical results when outliers are not present. For a shorter period including outliers, compared to the full sample, larger differences can be seen for data covering March 2020–June 2020. More specifically, the estimated variance of WTI error is 0.316 by VAR, which is over two times larger than those by RVAR.L (0.087) and RVAR.M (0.099). It is also worth noting that the estimated correlation between WTI and BRE errors reduces to 0.61 by VAR, whereas RVAR.L and RVAR.M still suggest estimates at around 0.85. Last but not least, contrasting diagonals (variances) of $\widehat{\varvec{\varSigma }}$ for RVAR.L or RVAR.M over the two sub-sample periods, only marginal differences are observed in most cases. This might suggest no structural changes across the two examined periods in terms of the model estimation. Deeper and more comprehensive analyses are performed for IRF and FEVD in subsequent sections.

**Table 6:** Estimated residual statistics

Model		July 2017–June 2020						July 2017–February 2020						March 2020–June 2020					
		XAU	XAG	BRE	WTI	CHF	JPY	XAU	XAG	BRE	WTI	CHF	JPY	XAU	XAG	BRE	WTI	CHF	JPY
VAR	XAU	0.121	0.089	0.034	0.034	0.045	0.060	0.119	0.085	0.029	0.030	0.044	0.062	0.094	0.075	0.032	0.038	0.041	0.032
	XAG	0.089	0.115	0.032	0.034	0.035	0.042	0.085	0.108	0.024	0.025	0.033	0.042	0.075	0.118	0.043	0.040	0.033	0.025
	BRE	0.034	0.032	0.121	0.116	0.018	0.034	0.029	0.024	0.109	0.102	0.011	0.027	0.032	0.043	0.131	0.124	0.048	0.049
	WTI	0.034	0.034	0.116	0.156	0.017	0.035	0.030	0.025	0.102	0.118	0.011	0.029	0.038	0.040	0.124	0.316	0.037	0.034
	CHF	0.045	0.035	0.018	0.017	0.092	0.061	0.044	0.033	0.011	0.011	0.089	0.058	0.041	0.033	0.048	0.037	0.109	0.074
	JPY	0.060	0.042	0.034	0.035	0.061	0.119	0.062	0.042	0.027	0.029	0.058	0.119	0.032	0.025	0.049	0.034	0.074	0.093
RVAR.L	XAU	0.114	0.084	0.024	0.026	0.039	0.053	0.108	0.077	0.020	0.021	0.037	0.053	0.082	0.071	0.017	0.023	0.022	0.015
	XAG	0.084	0.109	0.024	0.027	0.032	0.039	0.077	0.099	0.017	0.020	0.029	0.038	0.071	0.115	0.031	0.034	0.017	0.006
	BRE	0.024	0.024	0.098	0.094	0.012	0.022	0.020	0.017	0.093	0.089	0.007	0.016	0.017	0.031	0.077	0.070	0.029	0.024
	WTI	0.026	0.027	0.094	0.111	0.011	0.024	0.021	0.020	0.089	0.105	0.006	0.018	0.023	0.034	0.070	0.087	0.021	0.015
	CHF	0.039	0.032	0.012	0.011	0.089	0.056	0.037	0.029	0.007	0.006	0.085	0.052	0.022	0.017	0.029	0.021	0.092	0.050
	JPY	0.053	0.039	0.022	0.024	0.056	0.109	0.053	0.038	0.016	0.018	0.052	0.106	0.015	0.006	0.024	0.015	0.050	0.070
RVAR.M	XAU	0.116	0.086	0.026	0.027	0.041	0.054	0.112	0.080	0.022	0.023	0.040	0.056	0.094	0.077	0.027	0.036	0.034	0.026
	XAG	0.086	0.112	0.025	0.028	0.033	0.039	0.080	0.102	0.018	0.021	0.031	0.039	0.077	0.120	0.037	0.046	0.029	0.019
	BRE	0.026	0.025	0.101	0.097	0.013	0.024	0.022	0.018	0.096	0.091	0.008	0.019	0.027	0.037	0.080	0.075	0.036	0.031
	WTI	0.027	0.028	0.097	0.115	0.012	0.025	0.023	0.021	0.091	0.107	0.007	0.021	0.036	0.046	0.075	0.099	0.031	0.025
	CHF	0.041	0.033	0.013	0.012	0.089	0.056	0.040	0.031	0.008	0.007	0.085	0.053	0.034	0.029	0.036	0.031	0.104	0.065
	JPY	0.054	0.039	0.024	0.025	0.056	0.109	0.056	0.039	0.019	0.021	0.053	0.108	0.026	0.019	0.031	0.025	0.065	0.083

This table presents estimated residual statistics over three periods: July 2017–June 2020, July 2020–February 2020, and March 2020–June 2020. For the original VAR model, the estimates are sample variance-covariance matrix. For the RVAR.L and RVAR.M models, the estimates are the corresponding $\widehat{\varvec{\varSigma }}$ as described in Sect. 2

#### IRF and FEVD

As explained in Sect. 2, IRF measures the response of one sequence to the change of certain orthogonal shocks. Among the examined safe haven assets, since only WTI exhibits large outliers, it is of interest to examine the IRFs of all sequences to changes of WTI shocks.^5^

**Fig. 3:**
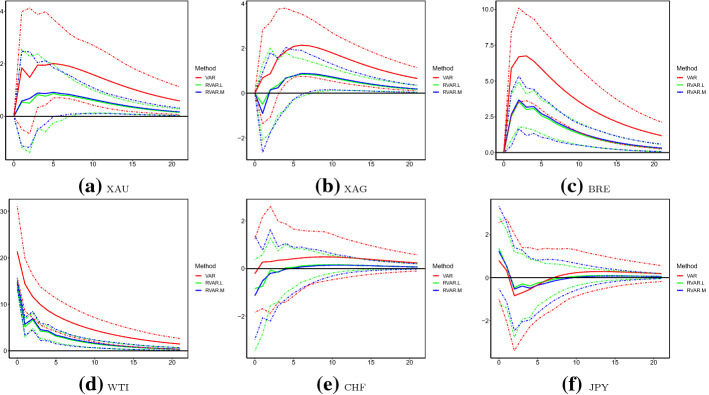
Impulse response functions with respect to WTI: July 2017–June 2020. *Note* This figure plots the impulse response functions (IRFs) of the six logged volatilities with respect to changes of WTI over July 2017–June 2020, produced using the original VAR model, robust VAR using the reweighted multivariate least trimmed squares estimator (RVAR.L) and robust VAR using the MM-estimator (RVAR.M). Solid lines are the mean estimates, and dashed lines are the corresponding 95% bootstrap confidence intervals produced with 1000 replicates. The unit of IRF is percentage in all cases

We firstly present our baseline results of the full sample. IRFs of the six sequences corresponding to WTI shocks are plotted in Fig. 3 up to 21 steps.^6^ To statistically compare the differences across employed models, we also provide the 95% bootstrap confidence intervals (CIs) using 1000 replicates.^7^ Recall that from Table 6, errors of WTI are strongly correlated with those of BRE, whereas the correlations between shocks of WTI and precious mental (currencies) are moderate (weak). From Fig. 3d, it can be seen that the contemporary orthogonal shock to WTI volatility is around 20% and 15%, estimated by the original and robust VAR models, respectively. As a response to this shock, BRE increases persistently, XAU and XAG are influenced only in at least one week, and currencies are not significantly affected. In terms of the magnitudes of changes, estimated IRFs (suggested by point estimates) of the original VAR model are almost uniformly higher than those of robust VAR models. Consistently with observations in Table 6 such that VAR estimates a larger variance of WTI shocks, CIs of VAR are much wider than those of RVAR.L and RVAR.M. This indicates the potential inefficiency of the original VAR model, when outliers are present in the dataset. Using the more efficient CIs of robust VAR models, it can be seen that IRFs estimated by the original VAR are significantly above those of the RVAR.L and RVAR.M for XAU and XAG over steps of 6 or above, and for BRE and WTI over steps of 1 or above. For instance, with a one-unit-standard-deviation change in the orthogonal shock of WTI, volatilities of XAU and XAG significantly increase by around 1% (2%) at step 6, according to the robust (original) VAR models. Also, RVAR.L and RVAR.M suggest that the maximum IRF of BRE to WTI is slightly above 3% at step 3, whereas that indicated by VAR is 7% also at step 3. Such significant differences may be attributed to large outliers of WTI over April 20–22, 2020 (Fig. 1).

**Fig. 4:**
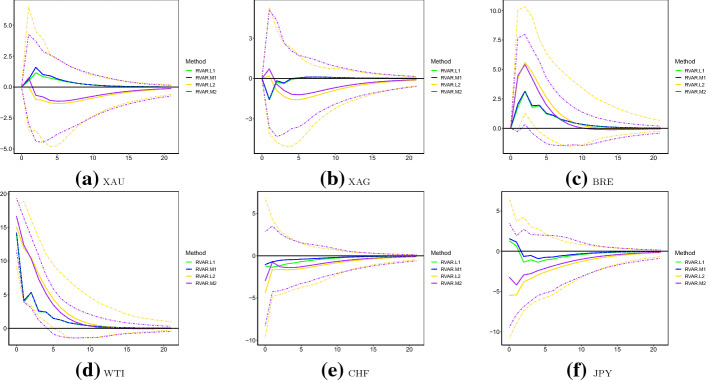
Impulse response functions with respect to WTI: July 2017–February 2020 vs March 2020–June 2020. Note: this figure plots IRFs of the six logged volatilities with respect to changes of WTI over July 2017–February 2020 and March 2020–June 2020, produced using the RVAR.L and RVAR.M models. Solid lines are the mean estimates, and dashed lines are the corresponding 95% bootstrap confidence intervals for data covering March 2020–June 2020. The numbers 1 and 2 in the legend indicate results over the first (July 2017–February 2020) and second (March 2020–June 2020) subsamples, respectively. The unit of IRF is percentage in all cases

Other than those larger outliers, from Fig. 2, we do observe relatively higher volatility levels after March 2020 than those over July 2017–February 2020. Consequently, one may question if IRFs have experienced structural changes over the two subsamples. If this is indeed the case, the uni-regime modelling framework examining the full sample might be problematic. However, due to the existence of larger outliers, results of the original VAR model are less reliable to investigate those potential structural changes. Therefore, we employ RVAR.L and RVAR.M for this aim. More specifically, we present point and interval estimates of IRFs over March 2020–June 2020 in Fig. 4. The point estimates of IRFs over July 2017–February 2020 are also plotted in the same figure. It can be seen that in all cases, point estimates of the first subsample consistently fall in CIs produced using the second subsample. This suggests that the sequence-specific IRFs over the two periods are statistically equivalent, and structural changes in IRFs may not exist. Nevertheless, it is worth noting that the widths of CIs produced by RVAR.L are wider than those of RVAR.M in some cases (e.g. BRE and WTI).

**Fig. 5:**
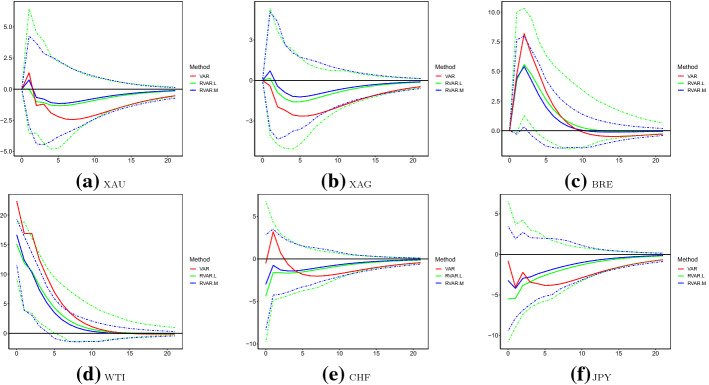
Impulse response functions with respect to WTI: March 2020–June 2020. *Note* This figure plots IRFs of the six logged volatilities with respect to changes of WTI over March 2020–June 2020, produced using the RVAR.L and RVAR.M models. Mean estimates of the original VAR model fitted by data with the three largest outliers (April 20–22, 2020) removed are also reported. The unit of IRF is percentage in all cases

Further, we check the validity/reliability of IRFs produced by the robust VAR models over March 2020–June, during which large outliers (over April 20–22, 2020) do exist. Specifically, we explore one modifications for the dataset: observations over April 20–22, 2020 are removed for all sequences. The modified samples are then fitted using the original VAR model. Since there are no abnormal observations in those cases, the results are much more reliable. Consequently, if RVAR.L and RVAR.M are valid in estimating IRF when outliers are present, the results should be comparable to those of the original VAR model using the modified samples. IRFs are plotted in Fig. 5, where VAR is fitted to the truncated data. In this case, we observe that point estimates of VAR fall in CIs of RVAR.L and RVAR.M at most steps across all sequences. Therefore, point estimates of VAR based on modified data are statistically equivalent to those of RVAR.L and RVAR.M for the original subsample, indicating their validity and reliableness.

Finally, we examine FEVD of each asset that is explained by orthogonal shocks of WTI. Consistent with IRF, steps up to 21 are considered, and we firstly discuss results of the full sample, which are presented in Table 7. Recall as defined in (4), FEVD is fully determined by estimates of variances of orthogonal shocks and IRFs. Thus, many of observations are similar to those of IRFs as described above. For instance, WTI shocks do not seem to explain much forecast errors of volatilities for currencies. Also, the original VAR model suggests that forecast errors of WTI volatility may be explained by its own orthogonal shocks at roughly 29% at step 1, with quite persistent magnitudes over subsequent steps. However, the RVAR.L and RVAR.M counterparties are 50% smaller and around 18% at step 1. Although still persistent, those FEVDs reduce to around 14% at step 20. Similarly, FEVD of BRE volatility to WTI shocks is around 10% according to the original VAR model, but is only 3% as estimated by RVAR.L and RVAR.M. Another distinct difference is the FEVD of precious metal. The original VAR implies that up to 2% proportion of forecast errors of XAU and XAG may be explained by WTI shocks in the longer term. In contrast, the corresponding FEVDs produced by RVAR.L and RVAR.M are only marginally different from 0. Again, with the existence of large outliers, FEVDs estimated via the robust VAR models are potentially more reliable.

**Table 7:** FEVD analysis: July 2017–June 2020

Model		Steps				
		1 (%)	5 (%)	10 (%)	15 (%)	20 (%)
VAR	XAU	0.0	0.8	1.6	2.0	2.2
	XAG	0.0	0.4	1.5	2.1	2.3
	BRE	0.0	7.3	10.1	10.8	11.1
	WTI	28.9	30.2	28.6	28.1	27.9
	CHF	0.0	0.0	0.1	0.2	0.2
	JPY	0.1	0.1	0.1	0.1	0.2
RVAR.L	XAU	0.0	0.1	0.3	0.4	0.4
	XAG	0.0	0.1	0.2	0.3	0.4
	BRE	0.0	2.2	3.1	3.2	3.3
	WTI	18.3	15.9	14.9	14.7	14.6
	CHF	0.1	0.1	0.1	0.1	0.1
	JPY	0.2	0.2	0.2	0.2	0.2
RVAR.M	XAU	0.0	0.1	0.3	0.4	0.4
	XAG	0.0	0.1	0.3	0.4	0.4
	BRE	0.0	2.4	3.3	3.5	3.5
	WTI	18.8	16.7	15.7	15.4	15.4
	CHF	0.1	0.1	0.1	0.1	0.1
	JPY	0.1	0.2	0.2	0.2	0.2

This table presents the FEVD explained by WTI shocks over July 2017–June 2020 of the three models: original VAR, RVAR.L and RVAR.M

**Table 8:** FEVD analysis: July 2017–February 2020

Model		Steps				
		1 (%)	5 (%)	10 (%)	15 (%)	20 (%)
VAR	XAU	0.0	0.6	0.7	0.7	0.7
	XAG	0.0	0.1	0.1	0.1	0.1
	BRE	0.0	2.3	2.7	2.7	2.7
	WTI	19.7	19.2	19.1	19.1	19.1
	CHF	0.0	0.1	0.1	0.1	0.1
	JPY	0.3	0.6	0.6	0.6	0.6
RVAR.L	XAU	0.0	0.2	0.3	0.3	0.3
	XAG	0.0	0.2	0.2	0.2	0.2
	BRE	0.0	1.5	1.7	1.7	1.7
	WTI	18.7	17.5	17.2	17.2	17.2
	CHF	0.2	0.6	0.7	0.7	0.7
	JPY	0.2	0.5	0.7	0.7	0.7
RVAR.M	XAU	0.0	0.4	0.4	0.4	0.4
	XAG	0.0	0.2	0.2	0.2	0.2
	BRE	0.0	1.6	1.9	1.9	1.9
	WTI	18.8	17.8	17.6	17.6	17.6
	CHF	0.1	0.2	0.2	0.3	0.3
	JPY	0.2	0.4	0.5	0.5	0.5

This table presents the FEVD explained by WTI shocks over July 2017–February 2020 of the three models: original VAR, RVAR.L and RVAR.M

**Table 9:** FEVD analysis: March 2020–June 2020

Model		Steps				
		1 (%)	5 (%)	10 (%)	15 (%)	20 (%)
VAR	XAU	0.0	1.2	2.9	3.5	3.7
	XAG	0.0	1.0	2.8	3.4	3.5
	BRE	0.0	17.1	17.0	16.9	17.0
	WTI	62.4	61.6	60.7	60.4	60.4
	CHF	0.6	1.2	3.0	3.5	3.6
	JPY	1.2	6.1	8.5	9.2	9.3
RVAR.L	XAU	0.0	0.3	0.7	0.8	0.8
	XAG	0.0	0.3	0.7	0.9	0.9
	BRE	0.0	4.9	5.3	5.3	5.3
	WTI	26.2	22.9	23.0	23.0	23.0
	CHF	2.1	2.2	2.6	2.7	2.7
	JPY	4.3	5.4	5.8	6.0	6.0
RVAR.M	XAU	0.0	0.2	0.4	0.5	0.5
	XAG	0.0	0.1	0.4	0.5	0.5
	BRE	0.0	4.4	4.5	4.4	4.4
	WTI	28.0	24.4	24.1	24.1	24.1
	CHF	0.8	0.9	1.2	1.2	1.2
	JPY	1.2	2.4	2.6	2.7	2.7

This table presents the FEVD explained by WTI shocks over March 2020–June 2020 of the three models: original VAR, RVAR.L and RVAR.M

**Table 10:** FEVD analysis: VAR results using truncated data

Assets	Steps				
	1 (%)	5 (%)	10 (%)	15 (%)	20 (%)
XAU	0.0	0.4	1.6	2.2	2.4
XAG	0.0	0.8	2.1	2.7	2.8
BRE	0.0	5.3	5.5	5.4	5.5
WTI	26.1	28.4	28.6	28.6	28.6
CHF	0.0	0.7	1.6	2.0	2.2
JPY	0.1	1.8	3.6	4.3	4.5

This table presents the FEVD explained by WTI shocks of VAR over the period containing large outliers: March 2020–June 2020. In the truncated sample, three largest outliers (April 20–22, 2020) are removed from the dataset

We now compare the two subsamples. FEVDs over July 2017–February 2020 are reported in Table 8, and those over March 2020–June 2020 are presented in Table 9. When there are no abnormal observations, we observe that the original and robust VAR models lead to much consistent results for data covering July 2017–February 2020. This suggests the reliability of RVAR.L and RVAR.M for data without outliers. For the second subsample (March 2020–June 2020), the original VAR argues that around 17% and 60% forecast errors of BRE and WTI volatilities can be persistently explained by WTI shocks. Those, again, are much greater than the robust counterparties, which are around 5% and 24% for BRE and WTI volatilities, respectively. Nevertheless, when following the approach analyzing IRF to discuss potential structural changes, FEVDs explained by WTI shocks do not significantly differ across the two subsamples. Taking the FEVD of WTI itself at step 1 as an example, the lower bound of 95% bootstrap CI of RVAR.L is 16.7% over March 2020–June 2020, smaller than the point estimate of 18.7% over July 2017–February 2020.^8^ Additionally, to check the validity of robust VAR results, we follow the strategy employed above and examine the two modified samples. The estimated FEVD using the original VAR model based on truncated sample over March 2020–June 2020 are reported in Table 10. It can be seen that those new results are largely consistent with robust estimates presented in Table 9, especially at larger steps. Therefore, the estimates of RVAR.L and RVAR.M models are expected to be reliable, and further inference can be made based on results of those methodologies.

#### Additional results and illustrative empirical implications

We now explore IRF and FEVD corresponding to the XAU and CHF shocks.^9^ The aim is to compare IRF and FEVD caused/explained by shocks of the precious mental futures and currencies to those of the oil shocks.

**Fig. 6:**
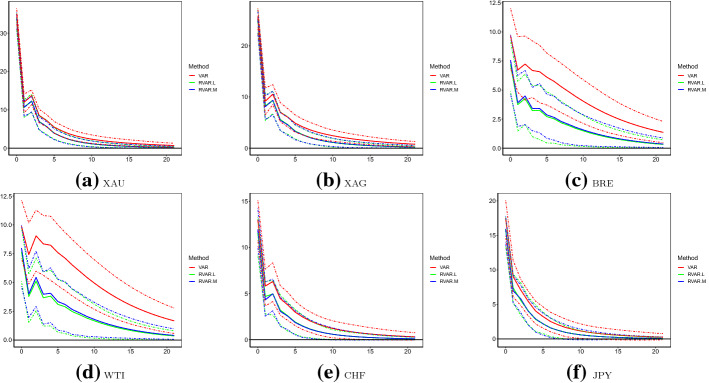
Impulse response functions with respect to XAU: July 2017–June 2020. *Note* This figure plots IRFs of the six logged volatilities with respect to changes of XAU over July 2017–June 2020, produced using the original VAR, RVAR.L and RVAR.M models. The unit of IRF is percentage in all cases

**Fig. 7:**
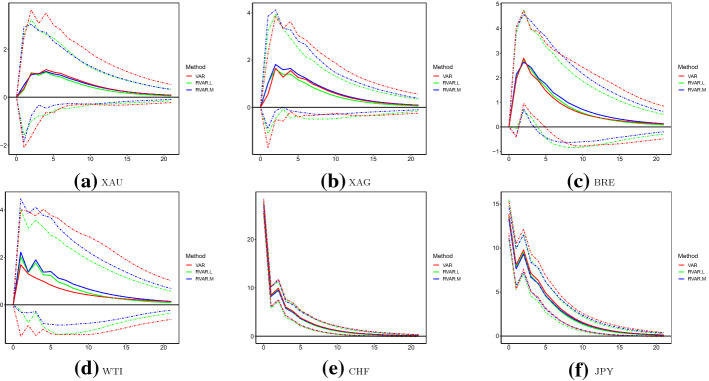
Impulse response functions with respect to CHF: July 2017–June 2020. *Note* This figure plots IRFs of the six logged volatilities with respect to changes of CHF over July 2017–June 2020, produced using the original VAR, RVAR.L and RVAR.M models. The unit of IRF is percentage in all cases

IRFs caused by XAU and CHF shocks are plotted in Figs. 6 and 7, respectively. Although no abnormally large outliers are observed for the logged XAU volatility, we observe that IRFs estimated by the original VAR are significantly larger than those robust counterparties. This conclusion holds at most steps of IRFs for XAU, XAG, BRE and WTI to shocks of XAU. In contrast, IRFs to CHF shocks across different models are statistically equivalent. This may be explained by the moderate (weak) correlations between XAU and WTI (CHF and WTI), as discussed above. Similarly, in Tables 11 and 12, we present FEVD explained by XAU and CHF shocks, respectively. Compared to the robust VAR models, it seems that the original VAR model overestimates the forecast errors explained by XAU shocks for the oil volatilities. For instance, FEVDs of BRE and WTI explained by XAU shocks are 15% (7%) at longer steps, as estimated by the original (robust) VAR model. FEVDs of other assets explained by XAU shocks and FEVDs of all assets explained by CHF shocks do not substantially vary across the original and robust VAR models.

**Table 11:** FEVD analysis: XAU

Model		Steps				
		1 (%)	5 (%)	10 (%)	15 (%)	20 (%)
VAR	XAU	100.0	95.2	91.1	89.5	88.9
	XAG	57.2	56.1	54.1	53.3	53.0
	BRE	7.7	12.3	14.8	15.7	16.0
	WTI	6.2	11.6	14.5	15.4	15.7
	CHF	18.2	20.9	21.3	21.4	21.5
	JPY	25.3	26.1	25.8	25.8	25.8
RVAR.L	XAU	100.0	95.5	92.5	91.6	91.4
	XAG	57.2	54.6	52.7	52.1	52.0
	BRE	5.3	6.3	6.7	6.8	6.9
	WTI	5.3	6.5	7.0	7.2	7.2
	CHF	15.0	16.1	16.1	16.1	16.1
	JPY	23.0	22.3	21.8	21.8	21.8
RVAR.M	XAU	100.0	95.7	92.5	91.5	91.3
	XAG	57.0	54.4	52.4	51.8	51.6
	BRE	5.7	6.8	7.2	7.4	7.4
	WTI	5.5	7.0	7.6	7.7	7.7
	CHF	16.1	16.6	16.5	16.4	16.4
	JPY	23.4	22.3	21.7	21.7	21.7

This table presents the FEVD explained by XAU shocks over July 2017–June 2020 of the three models: original VAR, RVAR.L and RVAR.M

**Table 12:** FEVD analysis: CHF

Model		Steps				
		1 (%)	5 (%)	10 (%)	15 (%)	20 (%)
VAR	XAU	0.0	0.2	0.4	0.4	0.4
	XAG	0.0	0.4	0.7	0.7	0.7
	BRE	0.0	0.9	0.9	0.9	0.8
	WTI	0.0	0.2	0.2	0.2	0.2
	CHF	81.5	74.9	72.9	72.4	72.2
	JPY	15.8	23.8	25.4	25.5	25.5
RVAR.L	XAU	0.0	0.2	0.4	0.4	0.4
	XAG	0.0	0.5	0.7	0.7	0.7
	BRE	0.0	1.2	1.4	1.4	1.4
	WTI	0.0	0.5	0.6	0.7	0.7
	CHF	84.6	79.7	78.5	78.3	78.2
	JPY	17.1	25.9	27.5	27.6	27.6
RVAR.M	XAU	0.0	0.2	0.4	0.4	0.4
	XAG	0.0	0.6	0.9	1.0	1.0
	BRE	0.0	1.2	1.5	1.5	1.5
	WTI	0.0	0.6	0.8	0.8	0.8
	CHF	83.5	78.6	77.3	77.0	77.0
	JPY	16.4	24.9	26.6	26.7	26.7

This table presents the FEVD explained by CHF shocks over July 2017–June 2020 of the three models: original VAR, RVAR.L and RVAR.M

The above results implies critical importance of the application of robust VAR analyses in operational practices. For instance, if one were to perform the original VAR analysis, the interaction between precious mental and oil futures may be overly stated. Consequently, inappropriate operational decisions could be made based on such analysis. For example, estimated impacts of XAU shocks on oil futures are upwardly biased. As a result, one may improperly adjust weights of oil futures in the portfolio, to hedge potential risks when a large XAU shock occurs. Such an action will likely lead to unwanted and/or ineffective risk mitigation results and cause unexpected loss.

We now compare robust IRFs caused by XAU, WTI and CHF, as described in above sections. It implies that orthogonal shocks of currencies cannot significantly increase volatilities of precious metal and oil futures. In contrast, orthogonal shocks of precious mental and oil futures can significantly influence each other, and those of XAU can significantly increase uncertainties of all safe haven assets. Also, significant responses to one-unit-standard-deviation shock of XAU are overall much larger than those to WTI and CHF shocks. Nevertheless, both IRF and FEVD suggest that changes in XAU may influence the precious mental futures and currencies more than the oil futures. Implications may also be related to the construction of portfolios. For example, XAU is still among the top allocations of safe havens (Baur & McDermott, 2016; Elie et al., 2019; Ji et al., 2020) in hedging risky assets such as the equity shares. If only safe haven assets were available to further mitigate risks of the idiosyncratic volatility of XAU, oil futures may be a more appropriate choice over currencies.

## Concluding remarks

This paper discusses the importance of employing robust vector autoregressive (VAR) models in the classic multivariate time series analysis with the presence of outliers. We focus on estimation consistency and efficiency of the residual variance-covariance ($\varvec{\varSigma }$) and popular metrics including the impulse response function (IRF) and forecast error variance decomposition (FEVD). Throughout this paper, two popular robust estimators: the reweighted multivariate least trimmed squares, or RMLTS, and multivariate MM-estimators are employed. The resulting robust VAR, or RVAR.L and RVAR.M models, are therefore adopted to illustrate the advantages of robust methodology over the classic ordinary least squares (OLS). Further, to select the appropriate number of temporal lags, we develop three robust counterparties of the popular Akaike information criterion (AIC), Bayesian information criterion (BIC) and Hannan–Quinn criterion (HQC).

In the simulation study, we show via various scenarios that while the investigated robust VAR models lead to rather similar results as the ordinary VAR in the absence of outliers, they produce much more accurate estimates in the presence of additive outliers. This conclusion holds for the inspected $\varvec{\varSigma }$, IRF and FEVD. Therefore, both RVAR.L and RVAR.M could be appealing alternatives to the original VAR model, when performing multivariate time series analysis and making relevant inferences.

Our empirical study is the first to examine the six popular safe haven assets considering the recent impacts of COVID-19 pandemic and the history-writing abnormal drops of the West Texas Intermediate (WTI) oil future. Specifically, the six assets include futures of gold (XAU), silver (XAG), Brent oil (BRE) and WTI oil and currencies of Swiss Francs (CHF) and Japanese Yen (JPY), all measured in US dollars. The daily realized volatilities over July 2017–June 2020 are examined. We find that the ordinary VAR model is severely influenced by large outliers (potentially over April 20–22, 2020) of WTI volatilities, and may produce overestimated variances of WTI residuals, and IRF and FEVD caused/explained by the orthogonal WTI shocks, compared to the robust VAR models. Further, when contrasting subsample results over July 2017–February 2020 (without outliers) and March 2020–June 2020 (with outliers) produced by RVAR.L and RVAR.M, we do not observe significant structural changes for IRF and FEVD corresponding to WTI shocks. Additionally, when the subsample of March 2020–June 2020 is modified to remove the impacts of outliers, the original VAR model provides statistically equivalent results to those of the robust VAR models based on the unmodified sample. Consequently, the validity and reliability of RVAR.L and RVAR.M to fit the full sample containing outliers are verified. Apart from WTI shocks, we further analyze IRF and FEVD related to XAU and CHF shocks. Our findings include significant ‘interdependence’ of the precious mental and oil futures (can impact each other), and ‘isolation’ of the currencies (cannot affect other assets). Also, orthogonal shocks of XAU are able to significantly influence volatilities of all examined safe haven assets. Therefore, critical empirical implications can be drawn. For instance, among the inspected safety assets, oil futures are more appropriate than currencies to mitigate risks of the idiosyncratic volatility of XAU.

It is worth noting some limitations of the proposed methods. For instance, those RVAR models might not be appropriate under the cellwise contamination (i.e., fully independent contamination model). In practice, this may occur when high volatility exists in some of assets at different periods. The reason why those RVAR models might not work well under this circumstance is that the breakdown point of affine equivariant estimators will decrease with the number of variables. A robust methodology which is designed for the fully independent contamination model remains for future works. Other future studies may consider extensions of the robust techniques in other contents. For instance, the seminal work of Primiceri (2005) discusses a time-varying VAR model and its application in examining monetary policies. Such a dynamic framework may also be constructed with all samples using time-varying weights (e.g., determined by a kernel method). As a result, without a robust methodology, the ordinary measure may suffer everlasting influences of a single outlier during the entire dynamic process. Extending the RMLTS and multivariate MM-estimators to those time-varying cases is therefore of critical importance for empirical analyses. Further, other robust estimators, including the Generalized S-estimator (Croux et al., 1994) and the Generalized M-estimator (Coakley & Hettmansperger, 1993), could be used to construct the robust VAR models that can be contrasted with RVAR.L and RVAR.M. In addition, this work focuses on the robust VAR to deal with the additive outliers, while robust methods that reduce the propagation effect from innovational outliers discussed in Muler (2013) and Muler et al. (2009) can be further investigated. Last but not least, the robust methods investigated in this paper focus on the case where the dimension of data *N* is relatively small and fixed. More recently, the case of high-dimensional scaling, in which the dimension *N* may increase to infinity with the sample size *T*, has become a popular area in time series analysis. The proposed models are therefore infeasible under such scenarios. To address this, recent studies have employed dimensionality reduction technique in the estimation (see, for example Qiu et al. 2015; Liu and Zhang 2021; Wang and Tsay 2021). It is of interest to extend those works to examine the effectiveness in estimation of IRF and FEVD, which are critical to empirical financial research. Those potential pathways remain for future works.

## Appendix: Computational packages

We use the software **R** to perform the computations of all models. VAR is implemented using the **vars** package, the RVAR.L and RVAR.M models are implemented using the functions that are written by the authors and based on **FRB** and **mlVAR** packages. The functions and codes to generate baseline simulation results can be found in https://osf.io/ehmbz/.

## Footnotes

1 Use the simulation sets described in Sect. 3.1, we perform analyses to study the proportions of those robust information criteria that correctly select the number of AR lags. The (robust) BIC outperforms the other two competitors when the (robust) VAR model(s) is employed, which validates our choice of (robust) BIC in the empirical analysis. Specific results are available upon request.

2 In addition, we have considered three modifications: 5% contamination rate instead of 1%; greater cross-regressive effects (0.6 instead of 0.3) than auto-regressive effects (0.3 instead of 0.6); and non-symmetric coefficient matrix (the second row of $\varvec{A}$ is set to (0.4, 0.5)). Materially identical conclusions can be drawn as those in Sects. 3.2 and 3.3. Specific results are available upon request.

3 We have also investigated the coverage rate of estimated $\varvec{A}$, based on 95% (bootstrap) confidence intervals. Robust VAR models outperform VAR with coverage rate much closer to 95% when outliers are present, whereas all coverage rates are comparable and close to 95% when no outliers are allowed. The results are available upon request.

4 Estimates of autoregressive coefficients are skipped here and are available upon request. Note that all fitted VAR system is covariance stationary. For instance, the smallest root of $|\det (\varvec{I}_N - \varvec{A}_1 z - \ldots - \varvec{A}_p z_p ) = 0|$ is 1.232 for the full sample, using the original VAR model.

5 Note that due to the feature of Cholesky decomposition, IRF and FEVD may depend on the order of series in a VAR system. In this paper, the six assets are included in the order of XAU, XAG, BRE, WTI, CHF and JPY. We also consider other orders, such as ranking WTI first or last, and the results are consistent and available upon request.

6 As a widely employed criterion, we assume that there are 252 trading days in each year, equivalent to 21 trading days in one month. Thus, 21 steps measure the responses up to one month for the daily data.

7 The bootstrap replicates are generated following the ordinary residual-based procedure. Specifically, bootstrap residuals are sampled with replacement from the original residuals. Bootstrap replicates are then equal to the summations of fitted values using the model based on original sample and bootstrap residuals. The 95% CIs consist of the corresponding 2.5% and 97.5% percentiles of an interested measure produced out of all bootstrap replicates.

8 Other detailed CIs are skipped here and are available upon request.

9 The results corresponding to XAG and JPY shocks are much consistent with those of XAU and CHF, respectively. IRF and FEVD of BRE shocks are similar to those of WTI shocks. Specific results are available upon request.
